# Unveiling the hidden allies of industrial chicory—a metagenomic exploration of rhizosphere microbiota and their impact on productivity and plant health

**DOI:** 10.3389/fmicb.2025.1509094

**Published:** 2025-05-09

**Authors:** Lalie Leclercq, Sony Debarre, Emily Lloret, Bernard Taminiau, Georges Daube, Caroline Rambaud, Djamel Drider, Ali Siah, Bruno Desprez, Jean-Louis Hilbert, Anca Lucau-Danila

**Affiliations:** ^1^UMRt BioEcoAgro 1158, University of Lille, JUNIA, INRAE, Univ. Liège, UPJV, Univ. Artois, Univ. Littoral Côte d’Opale, Villeneuve d’Ascq, France; ^2^Joint Laboratory CHIC41H University of Lille-Florimond Desprez, Cité scientifique, Villeneuve d’Ascq, France; ^3^University of Lille, IMT Lille Douai, University Artois, Junia, ULR 4515 – LGCgE, Laboratoire de Génie Civil et géo-Environnement, Lille, France; ^4^Department of Food Sciences, Microbiology, FARAH, University of Liege, Liege, Belgium; ^5^Florimond Desprez Veuve & Fils, Cappelle-en-Pévèle, France

**Keywords:** *Cichorium intybus*, root microbiome, metagenomics, *Streptomyces* ssp., *Penicillium* ssp.

## Abstract

**Background:**

As industrial chicory is significant for food, fodder, and medicinal purposes, its cultivation is increasingly crucial for producers. To enhance productivity, resistance, and the nutritional and functional values of this plant, we aimed to investigate its interactions with the microbial environment. We performed the first comprehensive taxonomic and functional characterization of the rhizosphere microbiota associated with industrial chicory, investigating how environmental factors influence its composition.

**Methods:**

Six different land plots were simultaneously cultivated with the same chicory genotype in northern France. Using soil analyses and metagenomic approaches, we characterized the diversity of bacterial and fungal communities in the soil microbiome associated with chicory plants and discussed their functional traits.

**Results:**

We observed significant taxonomic variability, influenced by soil composition and cultivation history across each plot. The presence of chicory plants distinctly shaped the microbial community. Specifically, chicory was found to recruit *Streptomyces* species that produce plant hormones and *Penicillium* species that facilitate phosphate solubilization and promote plant growth. Moreover, the plant demonstrated an ability to repel pathogens and adapt to local microbial communities by selectively favoring beneficial microorganisms according to local stresses and nutritional needs.

**Discussion:**

Our study represents a comprehensive taxonomic and functional analysis of the *Cichorium intybus* rhizosphere microbiome, underscoring the pivotal role of soil composition and land-use history. The specific microbial recruitment by chicory was also addressed.

## 1 Introduction

Industrial chicory is a cultivated plant well known for its food, fodder, and medicinal importance (Pouille et al., 2020; Fouré et al., 2018). Many plant metabolites have been identified as essential to confer their quality as a functional food (Pouille et al., 2022). Several agronomic and physiological parameters contribute to maintaining the expected metabolic composition of this plant. Among them, we must consider the root microbiome, which plays a vital role in plant growth and health. Very few studies have documented the composition of microbial communities associated with chicory. One microbiological study identified a limited number of taxa related to the roots of *Cichorium intybus* var. *foliosum* using *in vitro* cultures. These taxa included *Pseudomonas paucimobilis*, *Xanthomonas maltophilia*, *Agrobacterium radiobacter*, *Flavobacterium* spp., and some Gram-positive isolates such as *Bacillus* and *Streptomyces* spp. (Outryve et al., 1988). The microbiota of chicory leaves of *Cichorium intybus* has been recently studied and was found to be mainly composed of the phyla *Proteobacteria* (new taxonomy *Pseudomonadota*), *Firmicutes* (new taxonomy *Bacillota*), and *Actinobacteria* (new taxonomy *Actinomycetota*). The most abundant bacterial orders identified were *Enterobacteriales*, *Pseudomonadales*, *Rhizobiales*, *Sphingomonadales*, *Bacillales*, and *Burkholderiales* (Yum et al., 2023). A variation in the microbiota composition was also observed between the spring and summer seasons, attributed to different cultivation and environmental factors (Yum et al., 2023). A biostimulant effect of fungal culture filtrates obtained from *Chaetomium globosum* and *Minimedusa polyspora* on growth performance and metabolomic traits of chicory plants was also recorded (Spinelli et al., 2022), as well as the potential role of the arbuscular mycorrhizal symbiont *Funneliformis mosseae* in the improvement of chicory nutritional value (Pepe et al., 2022).

To approach a characterization study of the root microbiota of this crop plant, several aspects must first be considered. The microbial composition of a land plot depends on the soil composition (Schreiter et al., 2014), especially the amount and quality of available nitrogen (Estendorfer et al., 2017), but also the plant cover composition (Hartman et al., 2018). At the rhizosphere level, the diversity of microorganisms associated with plant roots is shaped by root architecture, exudates, and mucilage (Badri and Vivanco, 2009). Changes in chemicals, pH, and redox gradient between a given plant species and its microbial ecosystem lead to an increase in the abundance of several specific microbial taxa and their metabolic activity (Schmidt et al., 2011). For cultivated soils, these changes must be consistent, relatively homogeneous throughout the cultivated area, and closely dependent on the land-use history (Schlatter et al., 2020). Understanding the specifics of plant recruitment could henceforth enable us to conduct targeted microbiological studies and select beneficial microorganisms for the plant. A thorough knowledge of these microorganisms could then lead to developing solutions such as biofertilizers, biostimulants, or biocontrol agents to support cultivation (Calvo et al., 2014).

In this study, we conducted the first thorough taxonomic and functional characterization of the rhizosphere microbiome of chicory plants (*C. intybus* var. *sativum*) using metataxonomic analysis, that is, the taxonomic characterization of microbial communities based on amplicon sequencing of taxonomic marker genes such as 16S rRNA or ITS (Marchesi and Ravel, 2015). Six different land plots were cultivated simultaneously with the same chicory genotype in the same region of northern France. We specifically aimed to characterize (i) the diversity of bacterial and fungal communities in the soil microbiome associated with chicory plants and (ii) the relationships among the microbial communities and functional traits. Our study presented for the first time a comprehensive taxonomic and functional pattern of chicory rhizosphere microbiome and highlighted the role of the soil composition, land-use history, and the specific microbial recruitment by this plant.

## 2 Materials and methods

### 2.1 Plants and sampling

**Figure 1:**
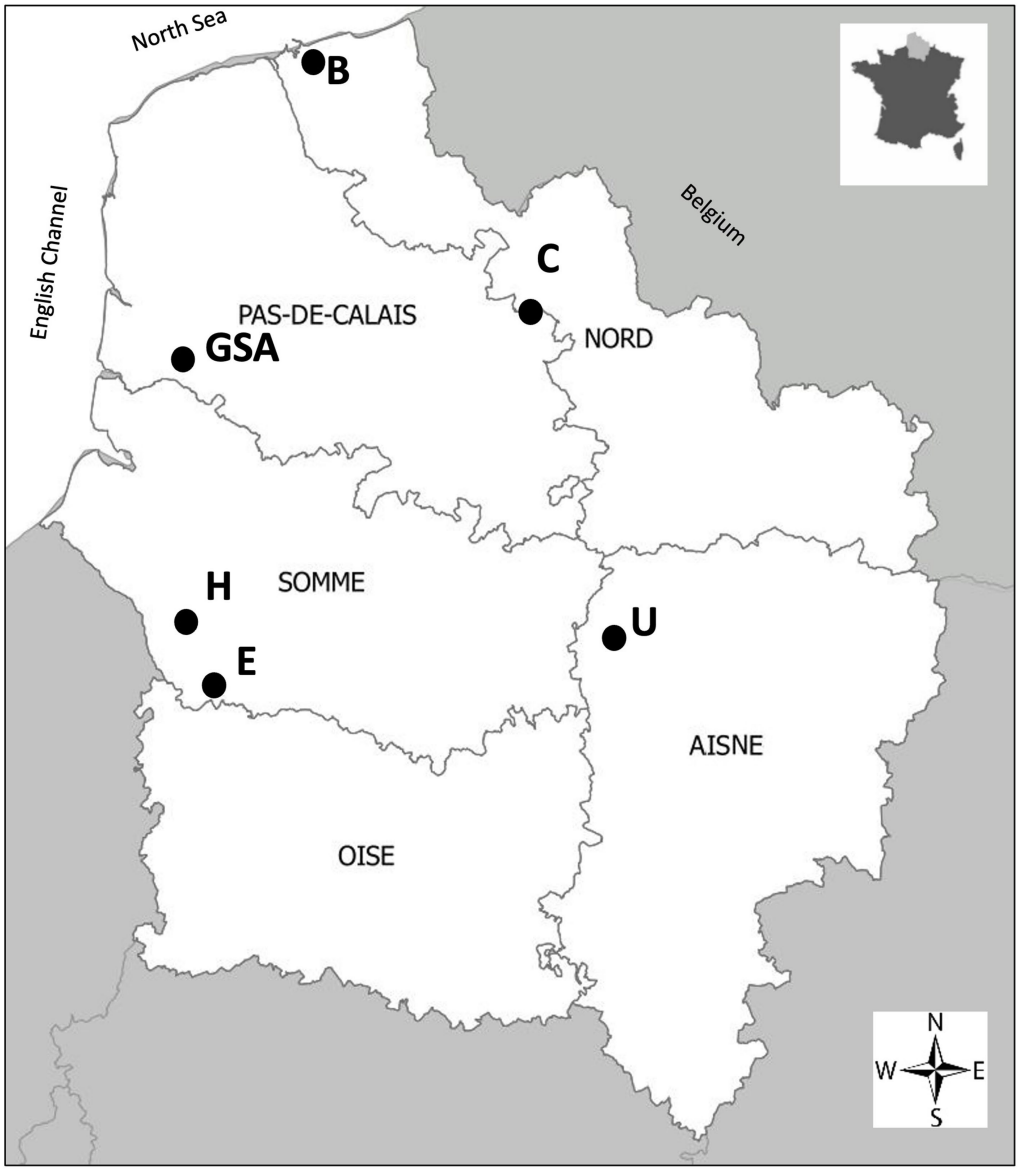
Map of northern France indicating the geographical position of the plots considered for analysis. Carvin (C), Brouckerque (B), Eplessier (E), Gouy-St-André (GSA), Hallencourt (H), and Urvillers (U).

Seeds of a single cultivar “Obsidienne” of the industrial chicory (*C. intybus* subsp. *intybus* var. *sativum*) were provided by Florimond Desprez Veuve & Fils (Cappelle-en-Pévèle, France). These seeds were grown as a monoculture under identical agronomic and organic farming conditions on six plots in northern France: Carvin, Brouckerque, Eplessier, Gouy-Saint-André, Hallencourt, and Urvillers plots (Figure 1). This region is characterized by a temperate oceanic climate, with a mean annual temperature of 10.2°C and a mean annual precipitation of 698 mm (data from Météo France). These six stands were selected in the same region to minimize climatic variation. Experiments were conducted in 2019. Samples were taken from plots characterized as clay–loam soil (Carvin, Guy-Saint-André), calcareous clay-loam soil (Brouckerque), and clay-limestone soil (Eplessier, Hallencourt, Urvillers).

The six plots presented a different cultivation history, with other species cultivated the previous year. The Brouckerque plot was fallow field the year before chicory was planted. Eplessier plot was also a fallow field, with white clover and ryegrass. Carvin plot was planted with wheat and then left fallow over the winter. Gouy-St-André plot was initially planted with sunflowers, and after harvest, a winter cover crop with a floral mix was sown. The Hallencourt plot was cultivated with wheat, followed by a winter cover crop containing red clover. Urvillers plot was planted with rapeseed and left fallow over the winter. No pesticides were used on the chicory plants, as all plots were managed as organic cultures. For soil analysis, five topsoil samples (0–15 cm of depth) were randomly collected in October 2019 from the field using a grid sampling method. Random coordinates within the grid were selected, ensuring a minimum distance of 10 between sampling points to capture representative soil conditions across the area. Approximately 500 g of soil was collected for each sample in clean, non-reactive polyethylene bags without additives. After collection, the samples were temporarily stored in a cool, dry environment until analysis.

For rhizosphere analyses, sampling was performed simultaneously, that is, in October 2019. Five random plants of each plot were pulled out of the soil, shaken gently to remove superfluous soil, and after roots were collected. Adhering soil was scratched and collected as a rhizosphere representative (Rhi). Five bulk soil samples (Ctrl) were randomly collected at least 2 m from any chicory plant, at bare soil level, at the depth corresponding to the chicory plants’ root (approximately 10–20 cm). Rhi and Ctrl samples were collected in sterile 50-mL Falcon tubes, freeze-dried, ground, and homogenized prior to DNA extraction.

### 2.2 Soil analyses

At least 10-20 g of soil was used to measure water pH (pH_water_), 5 mg for total carbon (TC), total nitrogen (TN) and total organic carbon (TOC), 1 g for mineral carbon analysis, and 5 g for major element analysis. Three replicates were performed for each analysis. To measure pH_water_, the soil samples were manually ground and sieved at 2 mm. A fraction of the soil below 2 mm was put in deionized water with soil:solution ratio of 2:5 (FAO, 2021) and stirred for 30 min. After decanting the soil, the pH was measured using a Hanna probe (HI 2211), with a precision of 0.05 pH unit. Two soil pH_water_ measurements were made: one with soil close to chicory roots, and another one with soil at a few centimeters away from chicory roots and representing the bulk soil.

For the other analyses, the soil samples were microground to below 10 μm. TC and TN concentrations were measured at the Laboratoire d’Océanologie et de Géosciences (LOG, Lille, France) using an elemental analyzer ThermoFisher Flash EA 1112 Series (Thermo Electron S.p.A., Milan, Italy), and TOC was analyzed with the same elemental analyzer after decarbonation of samples in caps with diluted concentrated HCl (one-fifth) and by calculation of the difference between TC and mineral carbon (CaCO_3_) measured by calcimetry using a De Bernard calcimeter (Jeulin, Evreux, France). The precision of these analyses is 0.05%. The TOC/TN ratio was also calculated. The mineralogical analyses were done at the Service d’Analyse des Roches et des Minéraux (SARM, Nancy, France) using an (Inductively Coupled Plasma – Optical Emission Spectrometry [ICP-OES] – iCap6500). The detection limits for SiO_2_, Al_2_O_3_, Fe_2_O_3_, MnO, CaO, Na_2_O, K_2_O, TiO_2_, and P_2_O_5_ are 0.050, 0.040, 0.015, 0.015, 0.030, 0.030, 0.020, 0.030, 0.020, and 0.100%, respectively. The loss on ignition (LOI) (Heiri et al., 2001) was also measured and corresponds to the mass difference between the sample mass before and after calcination.

### 2.3 Amplicon profiling

Total DNA from rhizosphere or control soil was extracted from five samples for each condition using the NucleoSpin DNA Stool Kit (Macherey-Nagel, Düren, Germany). DNA amounts were quantified by using BioSpectrometer (Eppendorf, Hamburg, Germany) and DNA quality was assessed with the 2100 Bioanalyzer (Agilent, Santa Clara, CA, US) (Supplementary material S1). The sequencing was done for each sample by the GIGA genoproteomic platform of Liège University (Belgium). For bacterial DNA sequencing, the amplification of the V1–V3 region of the 16S ribosomal DNA (rDNA) and the library preparation were performed with the following primers: direct (50-GAGAGTTTGATYMTGGCTCAG-30) and inverse (50-GAGAGTTTGGCTCAG-30). For fungal DNA sequencing, the amplification of the Internal Transcribed Spacer (ITS) regions 5.8S-ITS2, and the library were prepared for each sample using universal primers with Illumina Overhang adapters (Illumina, Evry, France) targeting the internal transcribed spacer 1 (ITS2) region. The forward primer ITS3KYO2 (5′-GATGAAGAACGYAGYRAA-3′), and the reverse primer ITS4 (5′-TCCTCCGCTTATTGATATGC-3′) were used for their broad coverage of fungal taxa. Single-organism DNA (*Escherichia coli* for bacteria and *Saccharomyces cerevisiae* for fungi) was used as a positive control. In contrast, a no-template control was used as a negative control to confirm the polymerase chain reaction (PCR) amplifications. Each PCR product was purified with the Agencourt AMPure XP Ball Kit (Beckman Coulter, Pasadena, CA, USA) and subjected to a second round of PCR for indexing, using Nextera XT index 1 and 2 primers. After purification, the PCR products were quantified using the Quant-IT PicoGreen (Thermo Fisher Scientific, Waltham, MA, USA) and diluted to 10 ng·μL^−1^. A final qPCR quantification of each library sample was performed using the KAPA SYBR FAST qPCR Kit (KapaBiosystems, Wilmington, MA, USA) before standardization, pooling, and sequencing on a MiSeq sequencer using v3 reagents (Illumina, San Diego, CA, USA). Data processing was performed using, respectively, the MOTHUR v1.44 package (https://mothur.org) and the VSearch algorithm (https://github.com/torognes/vsearch) (Rognes et al., 2016) for alignment, clustering, and chimer detection as previously described by Gérard et al. (2021).

After the cleaning process, sequences were clustered into operational taxonomic units (OTUs) at 97% of identity. Alignment and taxonomical identification were performed with MOTHUR using the SILVA v1.38.1 database (https://www.arb-silva.de/) of full-length 16S rDNA and the Unite database v9.0 (https://unite.ut.ee/) for 5.8S rDNA gene sequences. A rarefied table of 10,000 reads by sample was used for further analysis. Reads were finally aggregated into phylotypes at the phylum and genus taxonomic levels. All the biosample raw reads have been deposited at the National Center for Biotechnology Information (NCBI) and are available under BioProject accession number PRJNA1161587. Data obtained from next-generation sequencing (NGS) analysis were analyzed for the alpha-diversity with the Shannon index, and graphical representations were performed using GraphPad software (GraphPad, Prism 9.0, Windows, Inc., San Diego, CA, USA), and beta-diversity with the principal component analysis (PCA) using the FactoMineR package in R version 3.5.2.^1^ The average relative abundance across the five sequenced samples was calculated and used for graphical representations for each condition.

### 2.4 Functional characterization

Functional characterization was performed with RAST server,^2^ BacDive,^3^ and literature for bacterial species. FungiDB,^4^ Mycobank,^5^ and literature data completed functions for fungal species.

### 2.5 Statistics

Variations in pH, organic content, and elemental composition were assessed across the plots. For all these data, the Shapiro–Wilk test was employed to evaluate data normality, while Bartlett’s test was used to examine homogeneity of variances. Statistical differences were determined using ANOVA and Tukey’s *post hoc* tests (*p* < 0.05) for pairwise comparisons.

Data obtained from NGS analysis were analyzed for alpha-diversity using the Shannon index (SI) that combines richness and evenness, emphasizing the proportional abundance of OTUs (Shannon, 1948; Shannon and Weaver, 1949). PRISM 9 (GraphPad Prism 9.0, Windows, Inc., San Diego, CA, USA) was used to generate graphical representations of the SI and to assess statistical significance using Student’s test (*p* < 0.05). Beta-diversity was assessed to evaluate the dissimilarity in microbial community composition between samples (Gower, 1966), using PCA with the FactoMineR package in R version 3.5.2 (see text footnote 1). Phylotype data analyses were conducted by comparing the experimental groups with their respective controls, normality was determined by the Shapiro–Wilk test, and homogeneity of variances was measured by Bartlett’s test, significant differences were evaluated by the Student’s *t*-test (*p* < 0.05).

## 3 Results

### 3.1 Soil characterization of the six land plots

According to the “Référentiels Régionaux Pédologiques” (IGCS-RPP, GIS Sol) and the soil map on the Geoportail website,^6^ the dominant soils are established Brunisols (Cambisols in World Reference Base [WRB]) for the Carvin, Gouy-St-André, Eplessier and Hallencourt lands, Neoluvisols (Luvic Cambisols in WRB) for the Urvillers, and Thalassosols (Tidalic Fluvisols in WRB) for the Brouckerque. For the Eplessier and Hallencourt, it is possible to notice the presence of Calcosols close to the stand. Brunisols are soils formed on non-acidic parent geological material (like in the region on calcareous or chalk substratum). They can be the evolution of Calcosols, with structural horizons “S” (structure with fine aggregates) typical of alteration pedological processes with liberation of iron oxihydroxides, and clay particles. They can evolve into Neoluvisols (and then Luvisols) in this region of the northern France, where precipitation can accentuate leaching processes and where there are processes of clay illuviation, and it appears to be an “E” horizon. Thalassosols are typical soils developed on marine or fluvio-marine deposits. They are influenced by the water table, and tides influence its fluctuation. Thalassosols are typical of low-lying coastal plains. The Brouckerque land, being located close to the coast of the North Sea (Figure 1), has a risk of submersion during periods of high tides, and it is normal to find this typical soil for this stand. Very close to the location of the Brouckerque station (less than 1 km), we can also find Brunisols. We can thus observe a clear difference in the soil composition among the six locations analyzed.

### 3.2 Mineralogical and organic analyses of soil

The averages of pH_water_ of soil around roots and bulk soil, concentrations of TC, TN, TOC, and significant elements, and loss in ignition (LOI) are presented in Table 1. For all stands, the mean pH_water_ of bulk soils is close to the neutral pH and varies between 6.91 and 7.43. These pH values are typical for Thalassosols and Brunisols formed on calcareous or chalky rocks, where the pH is generally less than 7.5 under cultures (Référentiel pédologique, 2008). The most acidic soil was recorded in Gouy-Saint-André (pH 6.91).

**Table 1:** Averages of pH_water_, total carbon (TC), total nitrogen (TN), total organic carbon (TOC), major elements concentration, and loss in ignition (LOI).

Plot	pH_w_	pH_w_	TN	CT	TOC^a^	SiO_2_	Al_2_O_3_	Fe_2_O_3_	MnO	MgO	CaO	Na_2_O	K_2_O	Ti_2_O_5_	P_2_O_5_	LOI
	Ctrl	Rhi	Ctrl													
			%	%	%	%	%	%	%	%	%	%	%	%	%	%
B	7.10 ± 0.32 n = 4	6.98 ± 0.35 n = 5	0.09 ± 0.01 n = 3	1.73 ± 0.09 n = 3	0.73 ± 0.03 n = 3	81.90*^4^ ± 0.59 n = 3	3.91*^4^ ± 0.04 n = 3	1.40*^4^ ± 0.03 n = 3	0.02 ± 0.01 n = 3	0.39 ± 0.01 n = 3	4.01*^4^ ± 0.16 n = 3	0.53 ± 0.01 n = 3	1.14 ± 0.01 n = 3	0.19*^4^ ± 0.01 n = 3	0.16 ± 0.01 n = 3	6.66 ± 0.21 n = 3
C	7.30 ± 0.18 n = 5	6.89 ± 0.25 n = 5	0.12 ± 0.01 n = 3	1.75 ± 0.08 n = 3	1.47 ± 0.07 n = 3	77.16 ± 0.33 n = 3	8.11 ± 0.03 n = 3	2.97 ± 0.01 n = 3	0.07 ± 0.01 n = 3	0.62 ± 0.04 n = 3	0.91 ± 0.06 n = 3	0.98 ± 0.01 n = 3	2.12 ± 0.02 n = 3	0.75 ± 0.01 n = 3	0.22 ± 0.02 n = 3	6.14 ± 0.11 n = 3
E	7.43 ± 0.11 n = 4	7.20 ± 0.33 n = 5	0.13 ± 0.01 n = 3	1.35 ± 0.05 n = 3	0.94 ± 0.04 n = 3	77.11 ± 0.11 n = 3	8.30 ± 0.35 n = 3	3.12 ± 0.09 n = 3	0.10 ± 0.01 n = 3	0.54 ± 0.02 n = 3	1.10 ± 0.30 n = 3	0.88 ± 0.02 n = 3	1.86 ± 0.02 n = 3	0.78 ± 0.01 n = 3	0.16 ± 0.01 n = 3	6.44 ± 0.28 n = 3
GSA	6.91 ± 0.72 n = 4	6.29 ± 0.31 n = 5	0.11 ± 0.01 n = 3	1.10 ± 0.05 n = 3	0.81 ± 0.06 n = 3	81.00*^4^ ± 0.09 n = 3	7.08 ± 0.05 n = 3	2.35*^4^ ± 0.04 n = 3	0.07 ± 0.01 n = 3	0.46 ± 0.01 n = 3	0.63 ± 0.02 n = 3	0.95 ± 0.01 n = 3	1.90 ± 0.02 n = 3	0.73 ± 0.01 n = 3	0.18 ± 0.01 n = 3	4.68 ± 0.22 n = 3
H	7.34 ± 0.19 n = 4	7.01 ± 0.11 n = 5	0.18 ± 0.03 n = 3	1.91 ± 0.38 n = 3	1.55 ± 0.33 n = 3	77.78*^2^ ± 0.72 n = 3	7.48 ± 0.11 n = 3	2.87 ± 0.07 n = 3	0.08 ± 0.01 n = 3	0.45 ± 0.01 n = 3	0.89 ± 0.15 n = 3	0.82 ± 0.01 n = 3	1.56 ± 0.01 n = 3	0.69 ± 0.01 n = 3	0.12 ± 0.01 n = 3	7.70 ± 0.69 n = 3
U	7.16 ± 0.21 n = 5	6.81 ± 0.42 n = 5	0.15 ± 0.01 n = 3	1.62 ± 0.08 n = 3	1.27 ± 0.07 n = 3	75.92*^4^ ± 0.62 n = 3	8.49 ± 0.04 n = 3	3.27 ± 0.01 n = 3	0.08 ± 0.01 n = 3	0.63 ± 0.02 n = 3	1.00 ± 0.39 n = 3	0.96 ± 0.01 n = 3	2.04 ± 0.01 n = 3	0.82 ± 0.01 n = 3	0.18 ± 0.01 n = 3	6.88 ± 0.28 n = 3

*_2_ *p* < 0.01, *_4_ *p* < 0.0001 (ANOVA, Tuckey’s test).

Values are presented with standard deviation done on n replicates. C, Carvin; B, Brouckerque; E, Eplessier; GSA, Gouy-St-André; H, Hallencourt; U, Urvillers. Ctrl, control soil; Rhi, rhizosphere. pH_w_, pH_water_.

a Average of TOC were done on the two methods (cf. soil collection and analyses part). Each method was done on three replicates.

The mean pH_water_ of soils around roots is more acidic than the pH_water_ of bulk soils, and varies between 6.29 and 7.01. The lower difference between these two pH values is for the Brouckerque plot, with a value of 0.13, and the maximum is for the Gouy-St-André plot, with a value of 0.62.

For all stands, the mean TOC and TN contents in the bulk soils range from 0.73 to 1.55%, and from 0.09 to 0.18%, respectively. The lowest TOC and TN contents are found for the Brouckerque plot (0.73 ± 0.03% for TOC, and 0.09 ± 0.01% for TN) and are probably due to the presence of Thalassosol. For the other stands, TOC and TN contents are close to 0.81–1.55% and 0.11–0.18%, respectively. The average value of TOC/TN ratio, which is connected to microbial activity, decomposition, mineralization rates of soil organic carbon, and the cycle of soil carbon and nitrogen (Cotrufo et al., 2021), varies across all stands, with the highest value recorded in Carvin (12.50) and the lowest in Eplessier (7.27). These low values are typical for cultivated soils, which are generally approximately 8–15, because in cropland, soil organic matter can be rapidly decomposed or there is carbon and nitrogen uptake and keeping by plants which are harvested (Liu et al., 2016).

The loss on ignition for all samples (Table 1) is less than 8% (ranging from 4.47 to 8.26%), with the highest average value recorded in Hallencourt (7.70%) and the lowest in Gouy-St-André (4.68%). These results indicate that there is not much organic matter in the samples, as confirmed by TOC analyses (<2%, Table 1). Therefore, the results of the analysis of the major elements are significant. SiO_2_ was the most abundant oxide, with mean values varying between 76 and 82%, followed by Al_2_O_3_, with mean values between 3.9 and 8.5%, as well as Fe_2_O_3_, K_2_O, CaO–Na_2_O, and MgO, generally in decreasing order.

### 3.3 Microbial diversity

**Figure 2:**
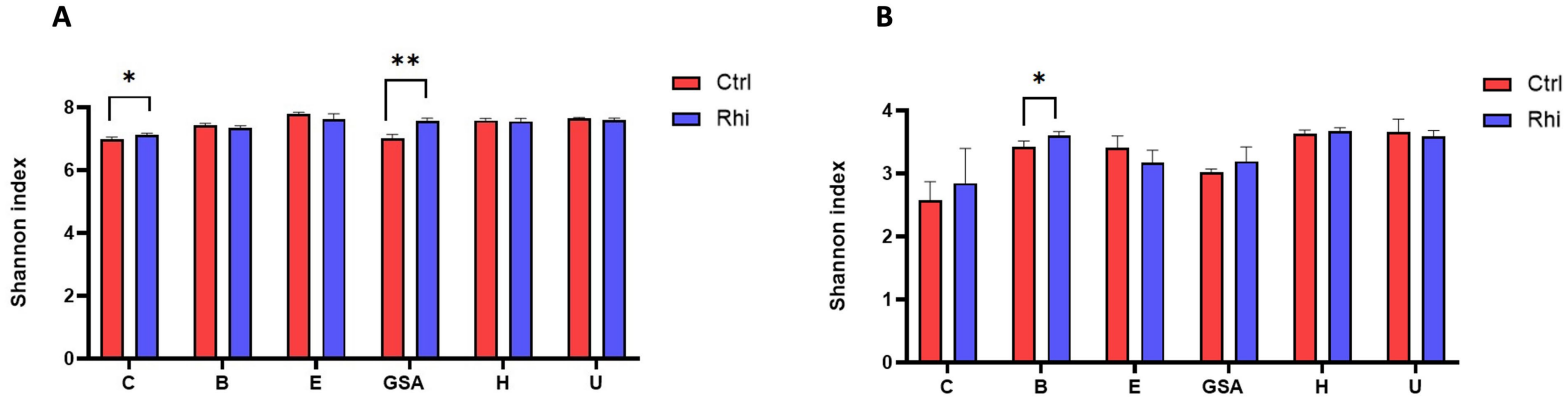
Microbial diversity of the bulk soil and rhizosphere of the six stations. Alpha-diversity of bacteria **(A)** and fungi **(B)** is illustrated by the Shannon index for each land plot: Carvin (C), Brouckerque (B), Eplessier (E), Gouy-St-André (GSA), Hallencourt (H), and Urvillers (U). Rhi, rhizosphere; Ctrl, bulk soil. *<0.05, **<0.01 (Student’s *t*-test).

The Shannon index representing the alpha-diversity was calculated to observe the changes in the diversity of bacteria and fungi. It provides information on the record of bacterial and fungal taxa at the root level and makes it possible to compare it with the bulk soil. We can observe that for the six land plots, there was a maintenance of taxa in the presence of cultivated plants, with a trend of greater diversity, with a Shannon index ranging between 6.5 and 7.8. This trend becomes significant for bacteria in Carvin and Gouy-St-André plots (Figure 2A), and for fungi in Brouckerque plot (Figure 2B), suggesting an increased microbial diversity at the rhizosphere level in these locations.

**Figure 3:**
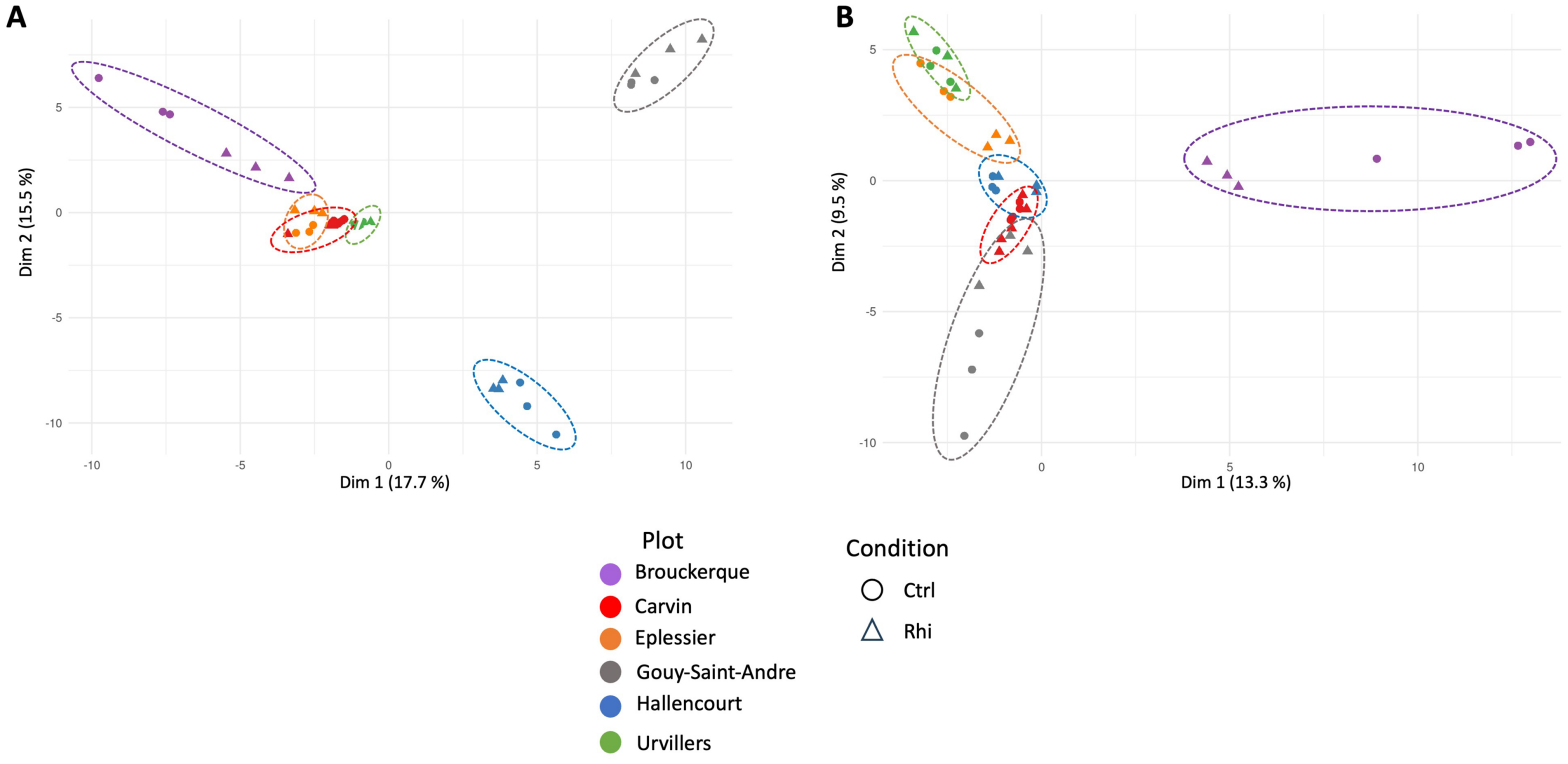
Principal component analysis representing beta-diversity for bacteria **(A)** and fungi **(B)**. Each point represents a replicate of bulk soil (Ctrl) or rhizosphere (Rhi) for each plot. Carvin (C), Brouckerque (B), Eplessier (E), Gouy-St-André (GSA), Hallencourt (H), and Urvillers (U).

To observe each land plot’s specific distribution of microbial OTUs, the beta-diversity was calculated for bacteria (Figure 3A) and fungi (Figure 3B). As each land plot had a particular soil composition and a specific cultivation history, differences in taxon distribution were remarkable. Brouckerque, Gouy-St-André, and Hallencourt plots stand out for bacterial taxa, Brouckerque and Gouy-St-André for fungal taxa.

### 3.4 Microbial abundance

**Figure 4:**
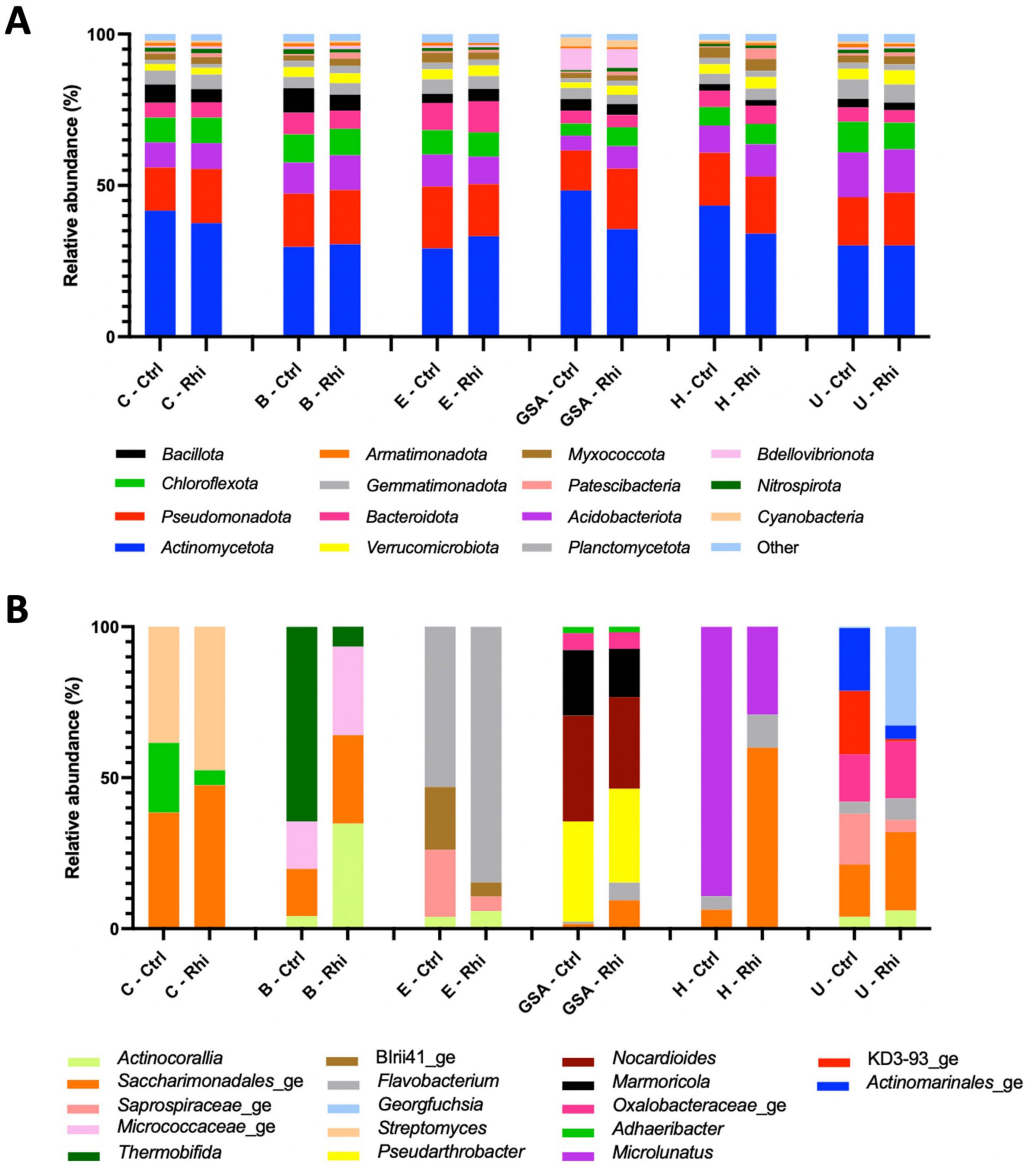
Bacterial composition of the bulk soil (Ctrl) and rhizosphere (Rhi) on the different land plots. Relative abundance of bacterial phyla **(A)** and genera **(B)** exceeding 1 and 10%, respectively, is represented for each land plot: Carvin (C), Brouckerque (B), Eplessier (E), Gouy-St-André (GSA), Hallencourt (H), and Urvillers (U).

**Figure 5:**
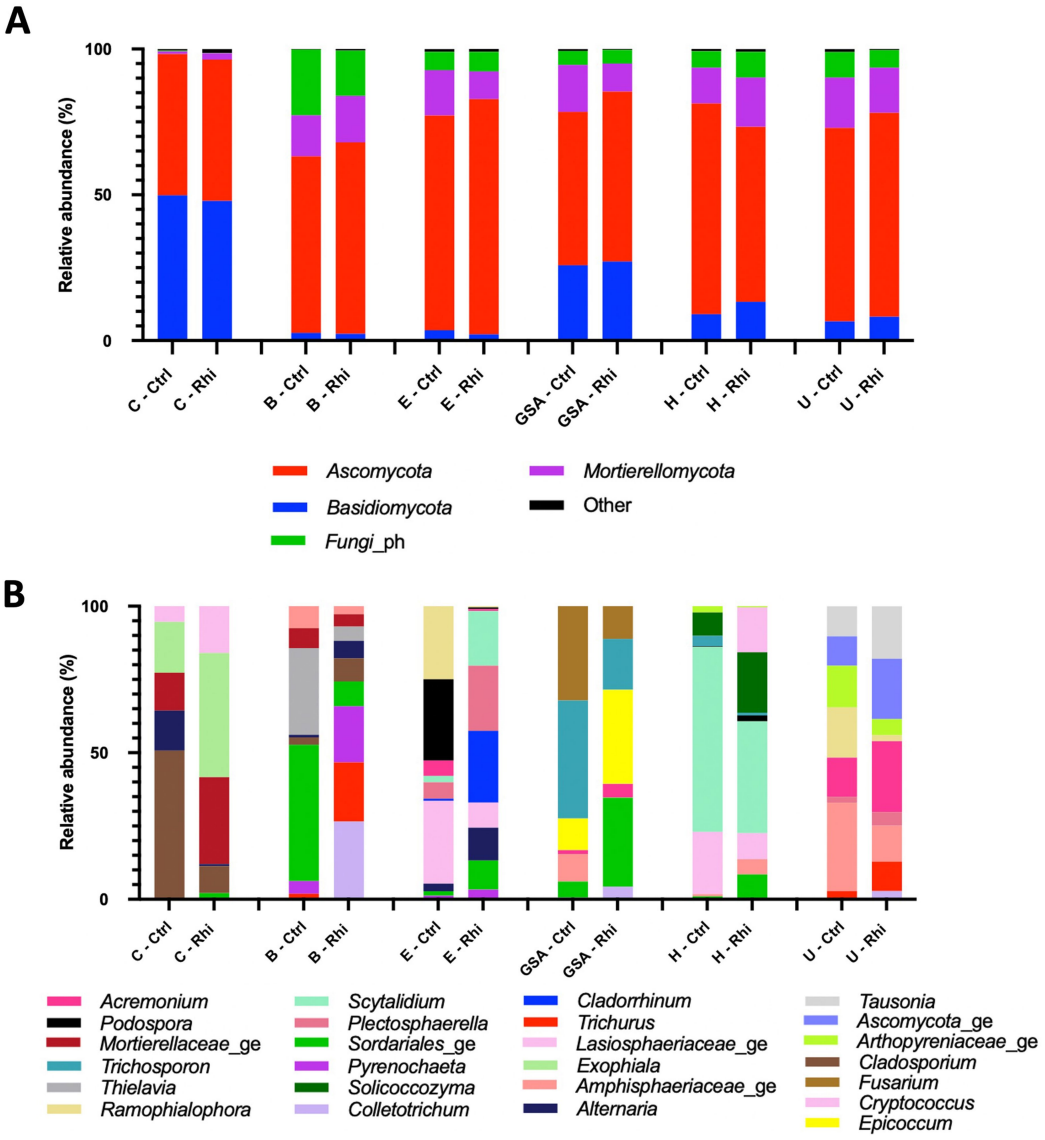
Fungal composition of the bulk soil (Ctrl) and rhizosphere (Rhi) on the different land plots. Relative abundance of fungal phyla **(A)** and genera **(B)** exceeding 1 and 10% respectively, is represented for each land plot: Carvin (C), Brouckerque (B), Eplessier (E), Gouy-St-André (GSA), Hallencourt (H), and Urvillers (U).

The relative abundance of phyla and microbial genera has been represented in Figures 4, 5. At the bacterial phylum level, differences among bulk soils of the 6 land plots were minor. The same 15 bacterial phyla were present (Figure 4A); *Actinomycetota* (between 30.1 and 37.7%) and *Pseudomonadota* (between 17.3 and 20%) being the most abundant, and no significant modifications in their relative abundance were recorded in the rhizospheres. At the level of fungal phyla, we observed the presence of the same four phyla across all samples, though variations among bulk soils appeared to be more significant. *Ascomycota* (58.3–80.6%) was found to be the most abundant phylum, except for the Carvin plot, where *Basidiomycota* reached 47.9% and was noticed as the majority phylum. As for bacteria, no significant variations in their relative abundance were recorded in the rhizospheres.

The genus relative abundance, in contrast, showed significant differences among the six plots, both for bacteria and fungi (Figures 4B, 5B). A large qualitative and quantitative variation was observed in bulk soil among the six plots (a field effect), most likely linked to each land plot’s composition and cultivation history. Looking at the abundance of bacteria or fungi in the rhizosphere, many quantitative and qualitative changes appeared compared to bulk soil (a plant effect).

**Figure 6:**
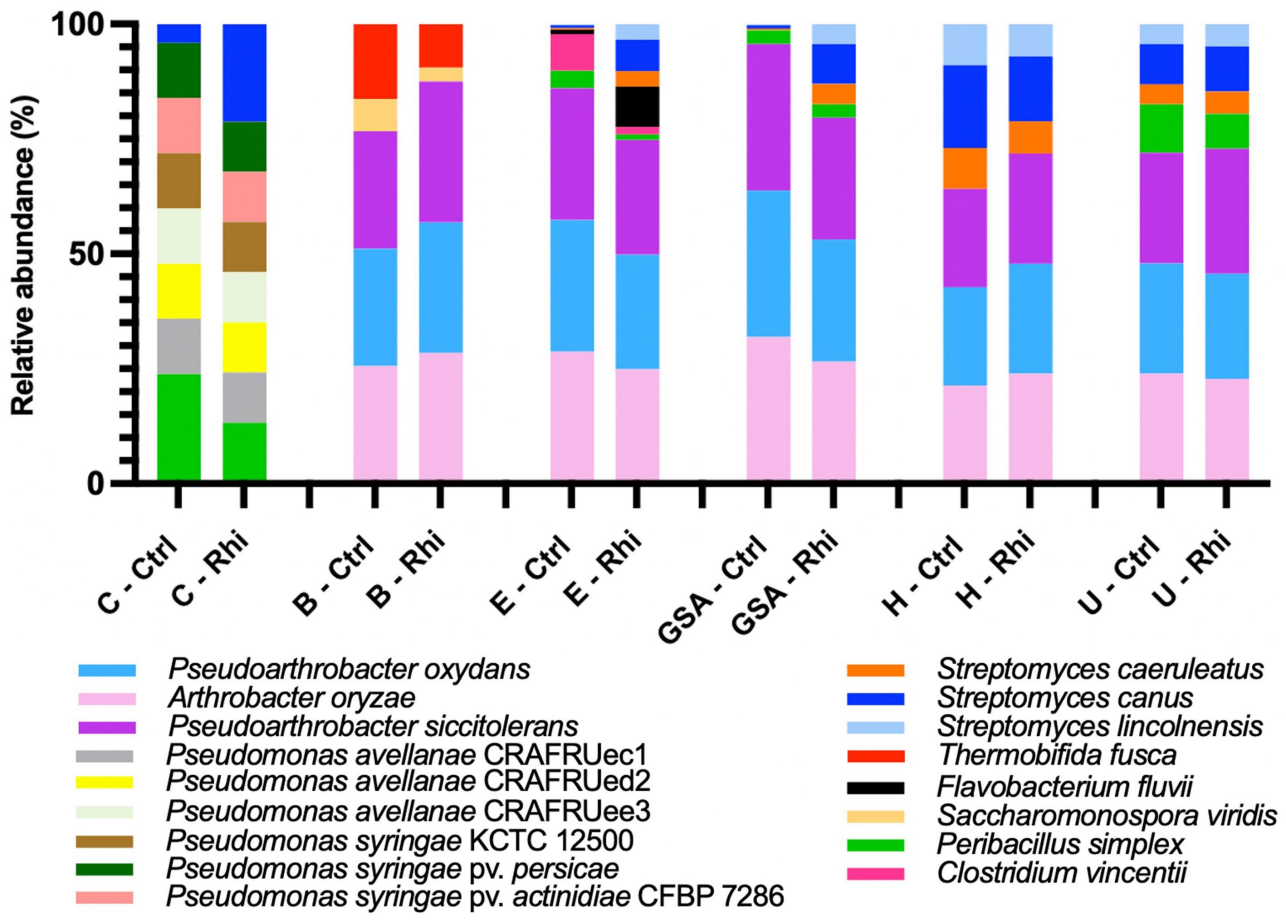
Abundance of annotated species of bacteria on bulk soil (Ctrl) and rhizosphere (Rhi) on the different land plots. Relative abundance of bacterial species exceeding 5% is represented for each land plot: Carvin (C), Brouckerque (B), Eplessier (E), Gouy-St-André (GSA), Hallencourt (H), and Urvillers (U).

**Figure 7:**
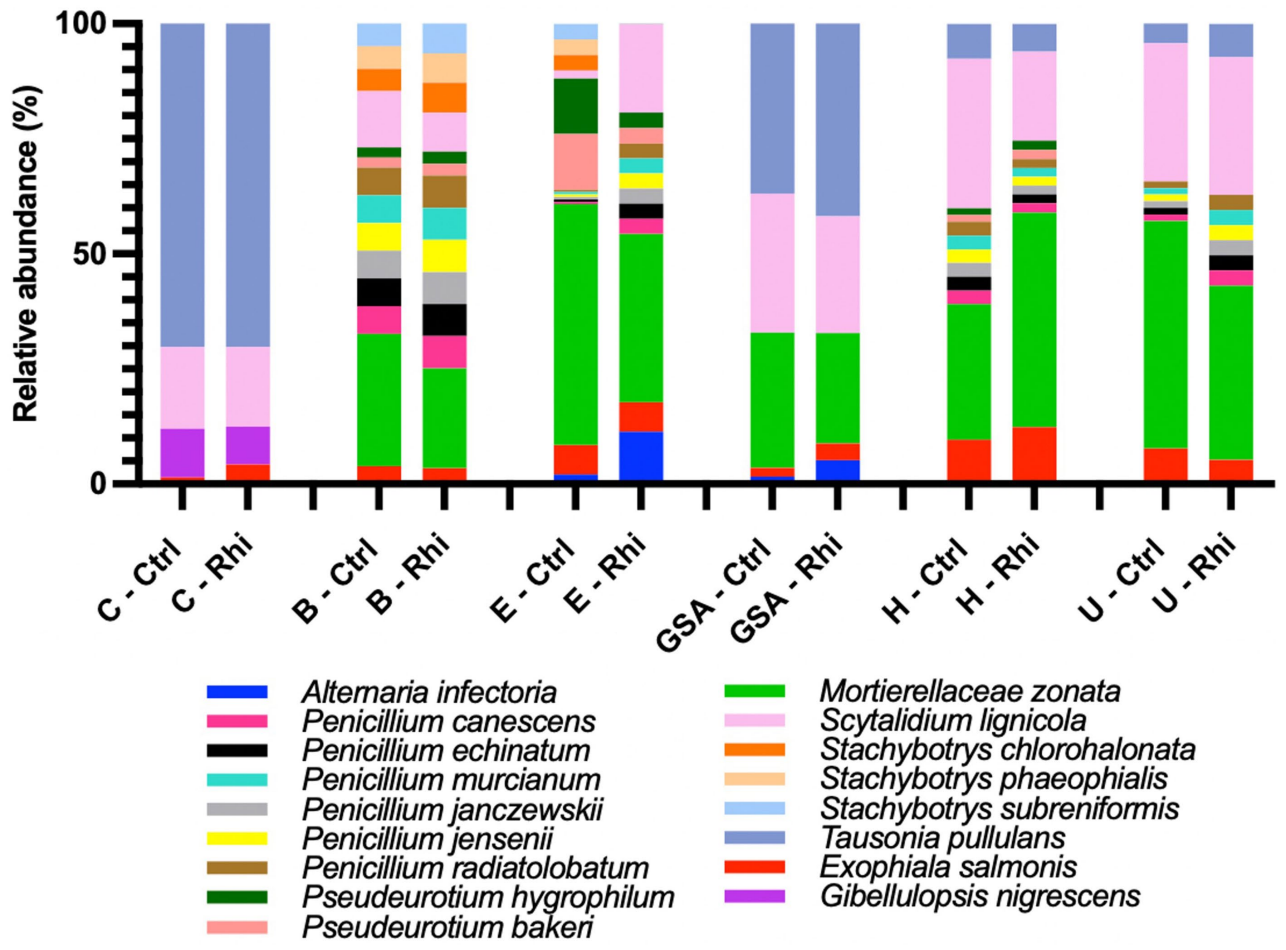
Abundance of annotated species of fungi on bulk soil (Ctrl) and rhizosphere (Rhi) on the different land plots. Relative abundance of fungal species exceeding 5% is represented for each land plot: Carvin (C), Brouckerque (B), Eplessier (E), Gouy-St-André (GSA), Hallencourt (H), and Urvillers (U).

At the species level, only 3.6% of the bacterial species and 61% of the fungal species identified were annotated using the SILVA v38.1 and Unite databases, and their abundances were evaluated. At the bacterial species level (Figure 6), we found that the Carvin plot significantly differed from the other plots. *Peribacillus simplex* (13.25%) and *Streptomyces canus* (21.21%) were the dominant species in the Carvin plot and *Arthrobacter oryzae* (22.8–28.4%), *Pseudarthrobacter oxydans* (22.8–28.4%) and *Pseudarthrobacter siccitolerans* (23.9–30.6%) in all the other plots. Concerning fungi, we found *Tausonia pullulans* as dominant in Carvin (70.2%) and Gouy-St-André (41.8%) plots, *Mortierellaceae zonata* (21.6–46.6%) for all other locations. *Scytalidium lignocola*, even present in all locations, was co-dominant in Gouy-Saint-Andre and Urvillers plots (25.4 and 29.8% respectively) (Figure 7).

Bacterial and fungal species associated with the rhizosphere or associated with control soil (ratio of relative abundance in rhizosphere vs. bulk soil above 1 or below 1) and significantly more abundant are represented in Supplementary material 2. For the six studied land plots, no significant common signature was observed at the taxonomical level.

### 3.5 Functional characterization of rhizosphere activity in the six land plots

**Figure 8:**
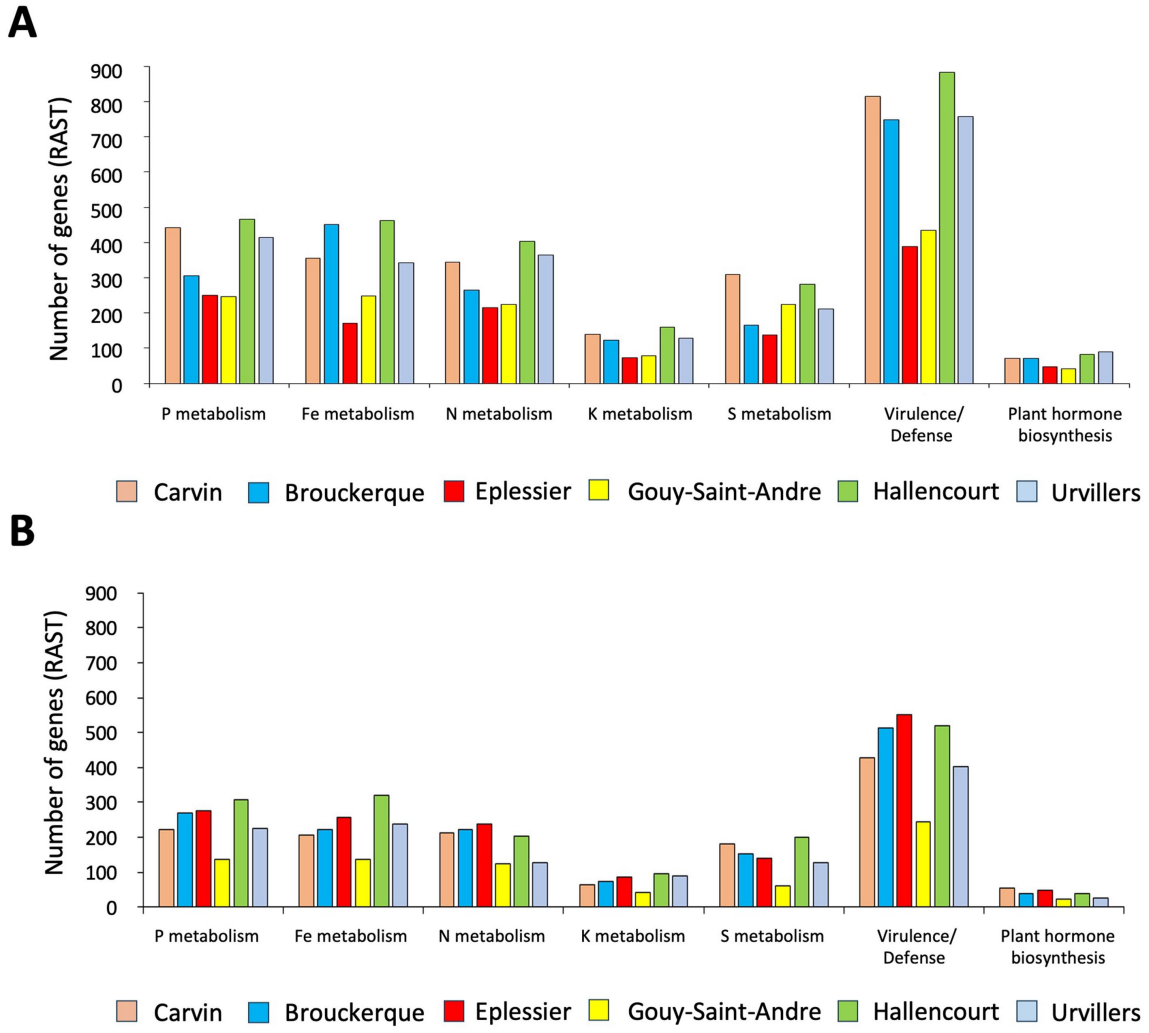
Number of bacterial genes involved in P, Fe, N, K, and S metabolism, virulence/defense, and plant hormone biosynthesis found associated with rhizosphere **(A)** or bulk soil **(B)** in the 6 land plots. Gene numbers were recorded according to RAST (https://rast.nmpdr.org/).

**Figure 9:**
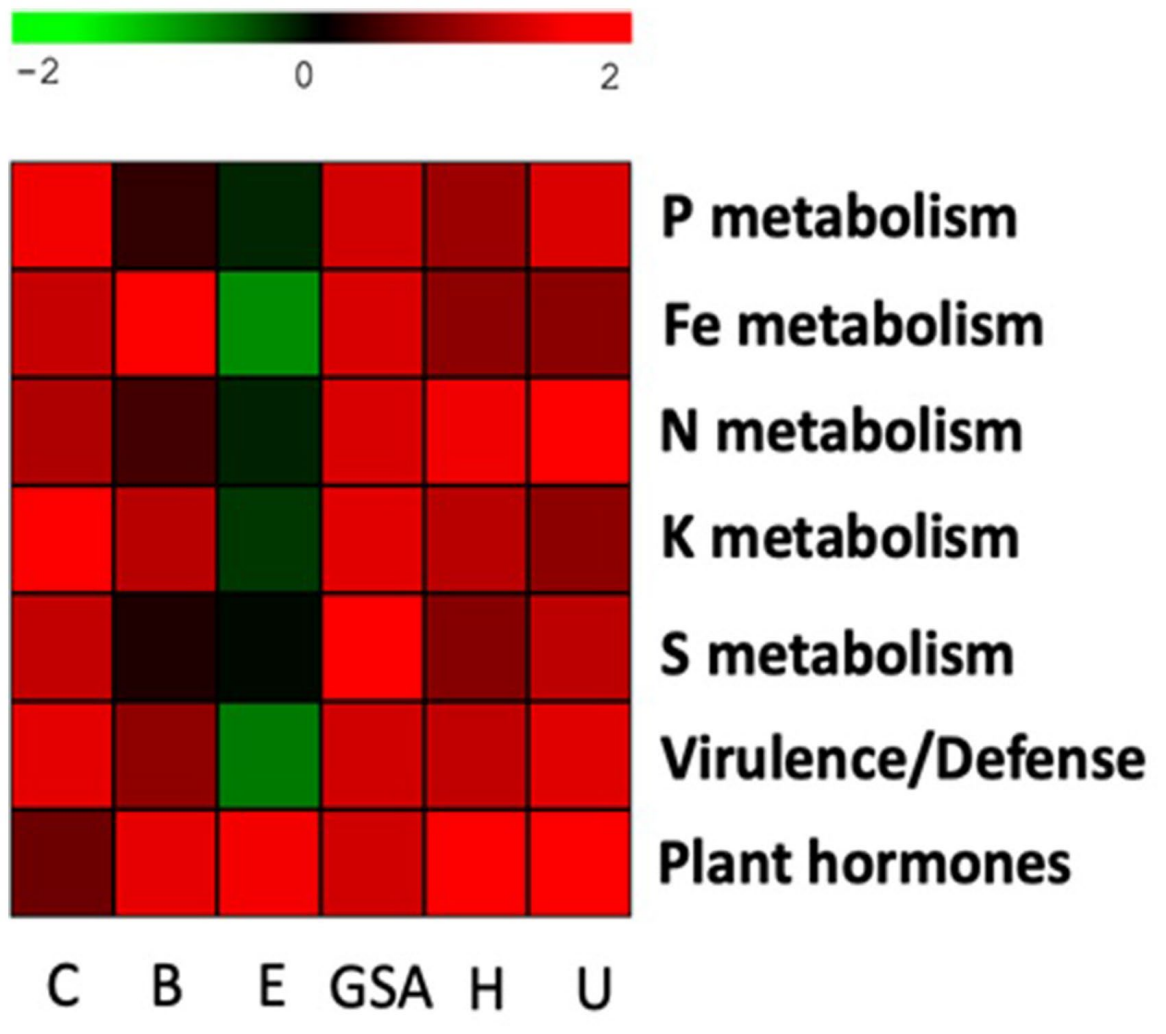
Heatmap with key functional pathways differentially represented in the rhizosphere and bulk soil. The log2 ratio between the number of genes in the rhizosphere and bulk soil was plotted for each station. Carvin (C), Brouckerque (B), Eplessier (E), Gouy-St-André (GSA), Hallencourt (H), and Urvillers (U).

Functional characterization of 3.6% annotated bacterial species was performed with the RAST fully automated service for annotating bacterial genomes (Figures 8, 9). To differentiate beneficial bacterial functions, we recorded the number of genes involved in P, Fe, N, K, and S metabolism and in virulence/defense and plant hormone biosynthesis. We observed a higher abundance of these genes in the rhizosphere (Figure 8A) compared to bulk soil (Figure 8B), suggesting that chicory plants recruit beneficial bacterial species. Eplessier and Gouy-St-André indicated that the less active rhizospheres, Hallencourt and Carvin, are more active for beneficial bacterial recruiting. For the same variety of chicory, the criteria for selecting bacteria should be based on nutritional needs (with soil composition being crucial) and the specific pathogens present in the environment (with cultivation history being a significant factor).

We calculated the ratio between the number of genes present in the rhizosphere and the number of genes in the bulk soil, and we represented it by a heatmap (Figure 9). We noted a variable recruitment of genes involved in the metabolism of P, F, K, S, and virulence and defense, from one station to another. The only common functional signature for all stations was the selection of bacteria possessing genes involved in producing phytohormones.

Concerning fungi, since we could not observe the gene counts of each species, we focused on commonly found species significantly linked to the rhizosphere across at least three stations. Using the FungiDB database and literature, we examined their functions. We identified fungi associated with the rhizosphere that are capable of phosphate solubilization, phytohormone production, and enzyme synthesis. Conversely, fungi avoided in the rhizosphere were predominantly known in the literature as phytopathogens (Table 2).

**Table 2:** Taxonomic and functional signature of bacterial species recruitment, associated with the rhizosphere (+) and bulk soil (−).

Origin of bacteria	Bacteria	Function	References
Rhizosphere associated bacteria (+)	*Streptomyces canus*	Antimicrobial activity	Odo et al. (2022)
	*Streptomyces turgidiscabies*	Cytokinin biosynthesis	Joshi and Loria (2007)
		Antimicrobial activity	
	*Streptomyces avidinii*	Antimicrobial activity	Teregulova et al. (2023)
	*Streptomyces lavendulae*	Antimicrobial activity	Jha et al. (2022)
	*Streptomyces spororaveus*	Antimicrobial activity	Chang et al. (2022)
	*Streptomyces virginiae*	Antimicrobial activity	Lang et al. (2023)
	*Streptomyces xanthophaeus*	Antimicrobial activity	Jeon et al. (2023)
Soil associated bacteria (−)	*Streptomyces pratensis*	Antagonist activity	Hu et al. (2023)
		Plant pathogen	Henao et al. (2023)
	*Streptomyces anulatus*	Antifungal activity	Kumar et al. (2023)

## 4 Discussion

As industrial chicory becomes increasingly essential for food, fodder, and medicinal purposes, its cultivation has gained significance among producers. To enhance productivity, resistance, and the nutritional and functional value of this plant, we aimed to explore its interactions with its microbial environment. Few studies have investigated the rhizosphere microbiota of chicory (Zeng et al., 2023) under abiotic stress conditions. In this study, we performed a comprehensive taxonomic characterization of the rhizosphere microbiota associated with industrial chicory and assessed how natural environmental factors and as well as agronomic practices influence its composition. Additionally, we performed a functional evaluation of the annotated species.

### 4.1 Soil composition, agricultural practices, and cultivation history

Six plots located in various regions of northern France were cultivated with the same chicory variety (Figure 1). For each plot, we analyzed the soil (Section 3.1) and the organic and mineralogical composition (Table 1). In all the plots, the high percentage of Si and Al, and the relatively high percentage of Fe compared to the other elements, indicated the presence of aluminosilicate minerals like clay minerals and iron oxihydroxides, resulting from the weathering of parent geological material and linked to the browning processes. The relatively high value of Ca found for the Brouckerque stand is probably due to the presence of carbonates. We would have expected higher levels of salts (Na and K) for this station due to saline conditions, but it is not the case, maybe due to the distance to the sea and the cropping activities. In contrast, except SiO_2_ and CaO, the Brouckerque stand presented the lowest mineralogical concentration compared to the others. It is well known that soil composition plays a crucial role in shaping the soil microbiome. Different soils supply varying amounts of organic and inorganic materials, leading to differences in nutrient availability and, consequently, influencing microbial growth and activity (Islam et al., 2020). Given the significant variability of these parameters across the six analyzed plots, it was not surprising to find highly diverse microbial communities at each station (Figure 3). Brouckerque, the only site characterized by a Thalassosol with an inferior elemental composition, stood out in terms of bacterial (Figure 3A) and fungal (Figure 3B) distribution. Another illustration of the interdependence between the soil and microbial diversity can be observed in the bacterial and fungal genera, which exhibit distinct compositions for each bulk soil in a plot-dependent manner (Figures 4B, 5B).

We also considered farming practices and cultivation history in the analysis of microbial diversity. It is well known that the soil cultivation history significantly influences the soil microbiome. Different crops and their cultivation practices can enrich specific microbial taxa while depleting others, reshaping the soil microbiome (Edwards et al., 2019). Considering the cultivation history of the different plots, we observed that the Gouy-St-André and Hallencourt plots, which share a similar soil (Neoluvisols) and have very close mineralogical and organic compositions (Table 1), were distinguished by their winter cropping history. These two plots exhibited highly distinct microbial profiles, particularly bacterial taxa (Figure 3A). Additionally, Gouy-St-André stood out for its fungal taxa composition (Figure 3B).

Regarding the agricultural regime, all plants were cultivated using the same technical itinerary for organic farming across all plots. However, the variations recorded in soil microbial composition could partly be linked to differences in soil nutrient levels. One of the hallmarks of organic farming is the use of amendments to replace synthetic industrial products (Mäder et al., 2002). These amendments, along with organic fertilizers, alter the soil’s composition and texture and exhibit variable transformation kinetics (Liang et al., 2006; Murphy, 2015), which can impact the metabolic conditions of microorganisms. Organic fertilizers are naturally derived materials from plant residues, animal manure, compost, and other organic matter. Unlike synthetic fertilizers, organic fertilizers release nutrients slowly (Guertal and Green, 2012) and do not provide all the necessary nutrients for plants (Havlin et al., 2014); instead, plants rely on nutritional supplements supplied on a case-by-case basis by the microorganisms present in each plot, depending on its specific cultivation history. Consequently, the organic cultivation method could lead to variations in the types of metabolisms developed by the soil’s microbial communities and implicitly in their taxonomic composition. Furthermore, differences in long-term agricultural practices applied specifically on each plot, such as crop rotation, continuous monoculture, or fallow, can result in shifts in the composition and function of soil microbial communities (Mayer et al., 2019).

### 4.2 Plant effect

Not only the soil but also the plant’s physiology can influence soil microbial communities, which is exerted through root exudates (Molefe et al., 2023). These root exudates contribute to nutrient cycling and enhance soil health by fostering symbiotic relationships between plants and microorganisms. In our experiments, the effect of the plant was reflected in the differences observed between the pH and the taxonomic composition of the control soil and that of the rhizosphere.

Regarding pH, we highlighted a systematic reduction in rhizosphere pH across all plots (Table 1). The plant effect on rhizosphere pH has already been well-documented. Roots can alter the pH of the rhizosphere through various mechanisms, including the release of organic acids and protons, the uptake of cations and anions, CO₂ dissolution from root respiration, and redox-coupled processes. This can lead to significant pH changes, influencing nutrient availability and microbial activity in the soil surrounding the roots (Nye, 1981; Hinsinger et al., 2003; Wei et al., 2024).

We can also assume that the higher acidity of the control soil in Gouy-Saint-André (pH 6.91) may be attributed to the activity of plants grown as a floral mix during the winter season (Table 1).

The effect of the chicory plant was also observed in the qualitative and quantitative changes in the rhizosphere, impacting both bacterial (Figures 2A, 4, 6) and fungal taxa (Figures 2B, 5, 7), as their diversity and composition differed from those of the bulk soil.

This effect has also been widely observed in other plant species, which shape microbial communities in the rhizosphere, affecting bacterial and fungal taxa (Philippot et al., 2013).

Since the microbial communities initially established in the different plots were highly diverse, we were unable to observe a specific taxonomic signature for chicory. However, in terms of species recruited and significantly associated with the plant’s roots, several species of bacteria (*S. canus*, *Streptomyces turgidiscabies*, *Streptomyces avidinii*, *Streptomyces lavendulae*, *Streptomyces spororaveus*, *Streptomyces virginiae*, and *Streptomyces xanthophaeus*) and fungi (*Penicillium canescens*, *Penicillium jensenii*, *Penicillium janczewskii*, *Penicillium echinatum*, *Penicillium murcianum*, and *Penicillium radiatolobatum*) were found in at least three out of six stations (Tables 2, 3).

**Table 3:** Taxonomic and functional signature of fungal species recruitment, associated with the rhizosphere (+) and bulk soil (−).

Origin of fungi	Fungi	Function	Reference
Rhizosphere associated fungi (+)	*Penicillium canescens*	P-solubilizing fungus	Mehta et al. (2019)
	*Penicillium jensenii*	P-solubilizing fungus	Castillo et al. (2013)
	*Penicillium janczewskii*	IAA producer	Murali et al. (2021)
	*Penicillium echinatum*	Cellulase producer	Martins et al. (2008)
	*Penicillium murcianum*	Cryptotanshinone producer	Chamkhi et al. (2022)
	*Penicillium radiatolobatum*	Antioxidant producer	Usman et al. (2024)
Soil associated fungi (−)	*Cladosporium angustisporum*	Plant pathogen	Prasannath et al. (2023)
	*Cladosporium anthropophilum*	Plant pathogen	Cho et al. (2023)
	*Cladosporium cladosporioides*	Plant pathogen	Li et al. (2024)
	*Cladosporium halotolerans*	Plant pathogen	Pouralibaba and Amirmijani (2021)
	*Cladosporium lycoperdinum*	Plant pathogen	Riascos et al. (2012)
	*Cladosporium perangustum*	Plant pathogen	Yang et al. (2021)
	*Cladosporium pseudocladosporioides*	Plant pathogen	Delisle-Houde et al. (2024)
	*Cladosporium subuliforme*	Plant pathogen	Ramos-García et al. (2016)
	*Cladosporium funiculosom*	Human pathogen	Sandoval-Denis et al. (2015)
	*Cladosporium inversicolor*	Human pathogen	Sandoval-Denis et al. (2015)

The plant effect, which could explain these changes as the chicory encounters a different bacterial community in each land plot and modifies it according to its needs, by recruiting or rejecting several taxa. The recruited or excluded taxa are not always the same, as the soil’s initial microbial composition varies from plot to plot. This leads us to believe that functional signatures may be more meaningful than taxonomic signatures.

### 4.3 Functional signature of the chicory root microbiota

To identify a functional signature of the annotated microorganism species, we analyzed the number of bacterial genes involved in metabolic pathways of interest for plant growth and resistance (P, Fe, N, K, and S metabolism, virulence/defense, and phytohormone biosynthesis) (Figures 8, 9). Additionally, we consulted databases (BacDive, FungiDB, Mycobank) and literature (Tables 2, 3) to target the main functional traits of each bacterial and fungal species. We observed variable recruitment from one station to another. On the Brouckerque and Eplessier plots, we observed a lower recruitment of bacteria capable of metabolizing various elements (phosphorus, iron, nitrogen, potassium, and sulfur). This can be explained by the soil being poorer in these elements, which would not favor the development of such bacteria (Table 1). Indeed, the soil of the Brouckerque plot is the poorest in nitrogen (0.09%) and among the poorest in phosphate (0.16%). Similarly, the soil of the Eplessier plot is also among the poorest in nitrogen (0.13%), phosphate (0.16%), iron (3.12%), and potassium (1.86%).

However, a direct link cannot be made between the soil composition and the presence of bacteria capable of metabolizing these elements; we must also consider the cultivation history of these plots. The Brouckerque and Eplessier plots were the only two plots left fallow the year before the chicory planting, which could explain their more impoverished microbial composition. Research conducted over 5 years demonstrated that fallow tillage affects soil microbial communities, often resulting in less diverse and functionally impoverished microbial populations than planted plots (Zhong et al., 2023). Fallow plots tend to show different community structures than planted plots, often having lower microbial diversity and altered compositions due to the lack of continuous organic inputs and plant-microbe interactions (Li et al., 2020). However, this state should not be considered irreversible; set-aside management following tillage can contribute to the gradual restoration of soil biodiversity (Żarczyński et al., 2023). Additionally, even though the functional signature of bacteria in soils with low availability of specific nutrients is different from that of nutrient-rich soils, it is common to find microorganisms capable of supporting plant nutrition by making these elements more accessible through various mechanisms. For example, nitrogen-fixing bacteria convert atmospheric nitrogen into a usable form for plants (Galloway et al., 2008), while phosphate-solubilizing bacteria enhance phosphorus availability by releasing organic acids that dissolve insoluble phosphate compounds (Bargaz et al., 2021). Additionally, mycorrhizal fungi form symbiotic relationships with plant roots, facilitating phosphorus uptake and other nutrients in nutrient-deficient soils (Smith and Read, 2008).

We observed variations in the recruitment of bacteria with antagonistic properties (Figure 9). Virulence and defense-related genes in antagonistic microbes typically include those involved in producing antimicrobial compounds such as bacteriocins, antibiotics, and siderophores, which help inhibit or outcompete pathogens. These genes can be classified into various categories, including those involved in quorum sensing, antibiotic biosynthesis, and the secretion of lytic enzymes that degrade pathogen cell walls (Ali et al., 2022; Lugtenberg and Kamilova, 2009) While virulence genes are typically associated with pathogenic bacteria, their presence in non-pathogenic microbes is not uncommon. These genes can provide competitive advantages in the microbial community and may reduce the pathogenic potential of these microbes under certain environmental conditions (Negi et al., 2023; White et al., 2019).

In our analyses, the Eplessier plot appears to have the microbial community with the fewest genes involved in virulence and defense (Figure 9). This could be explained by the unique cultivation history of plots, which favors particular microbial communities. We can therefore suggest that on the Eplessier plot, the chicory encountered a microbial community that did not require much antagonism. The only common functional signature observed across all plots was the selection of bacteria possessing genes involved in producing phytohormones (Figure 9). These genes are typically related to synthesizing secondary metabolites, such as phytohormones (e.g., auxins and gibberellins), and contribute to plant growth promotion (Amara et al., 2015).

In the fungi recruited by the chicory rhizosphere, we found species solubilize phosphate and produce phytohormones and enzymes. Remarkably, many species known as pathogens are found significantly more abundant in bulk soil (Table 2).

These recruitment trends are described in the literature for other species, such as *Arabidopsis thaliana* or tomato. Indeed, various signaling mechanisms in the rhizosphere can influence the recruitment of plant hormone-producing microorganisms (Venturi and Keel, 2016; Berendsen et al., 2018), P-solubilization (Alori et al., 2017), or the pathogens’ avoidance through interactions with beneficial microorganisms and activation of defense mechanisms (Mendes et al., 2013; Berendsen et al., 2012).

We have identified a few *Streptomyces* (*S. canus*, *S. turgidiscabies*, *S. avidinii*, *S. lavendulae*, *S. spororaveus*, *S. virginiae*, and *S. xanthophaeus*), and *Penicillium* species (*P. canescens, P. jensenii*, *P. janczewskii*, *P. echinatum*, *P. murcianum*, and *P. radiatolobatum*) species associated with the rhizospheres of industrial chicory that are involved in the different functional signatures. These species will be considered for future targeted microbiological studies aimed at developing biocontrol solutions for the cultivation of this plant.

In conclusion, our analysis of the environmental impact on the rhizosphere microbiota composition of industrial chicory revealed significant taxonomic variability associated with soil composition, agricultural practices, and the cultivation history of each studied station. The host plant was also found to exert a pronounced effect, actively recruiting and shaping the microbial community associated with its roots. We did not observe a significant common taxonomic signature in the chicory rhizospheres; our findings suggest a functional recruitment process influenced by the pre-existing microbiological composition. Functionally, chicory exhibits an inclination for *Streptomyces* species that produce plant hormones and *Penicillium* species that enhance phosphate solubilization and promote plant growth. Moreover, chicory demonstrates the ability to repel pathogens and adapt to local microbial communities by selectively favoring beneficial microorganisms that address local nutritional needs and environmental stresses.

## Data Availability

The dataset for this study can be found in the NCBI repository with the accession number PRJNA1161587.

## Footnotes

^1^ https://posit.cloud/

^2^ https://rast.nmpdr.org/

^3^(https://bacdive.dsmz.de)

^4^(http://fungidb.org/)

^5^(https://www.mycobank.org)

^6^ www.geoportail.gouv.fr
