# Supplementary material for: Unveiling the hidden allies of industrial chicory—a metagenomic exploration of rhizosphere microbiota and their impact on productivity and plant health
Source: Front Microbiol. 2025 May 9;16:1509094. doi: 10.3389/fmicb.2025.1509094 (PMC12098591; doi:10.3389/fmicb.2025.1509094)
Supplement: Supplementary file 1 [file Data_Sheet_1.zip › Supplementary Material 2.docx]

|  | **Carvin** | **Brouckerque** | **Eplessier** | **Gouy-Saint-Andre** | **Hallencourt** | **Urvillers** |
| --- | --- | --- | --- | --- | --- | --- |
| **Rhizosphere associated bacteria** | *Actinosynnema mirum*  *Arthrobacter crystallopoietes*  *Nocardia globurela*  *Nocardioides albus*  *Nocardioides luteus*  *Nonomuraea glycinis**  *Pseudomonas avellanae*  *Pseudomonas migulae*  *Pseudomonas mohnii*  *Pseudomonas syringae*  *Rhodococcus erythropolis*  *Rhodococcus globerulus*  *Streptomyces althioticus*  *Streptomyces canus***  *Streptomyces europaeiscabie***  *Streptomyces mutomycini*  *Streptomyces turgidiscabies**  *Variovorax paradoxus* | *Bacillus aryabhattai*  *Bacillus cereus*  *Bacillus megaterium*  *Bacillus mycoides*  *Bacillus subtilis*  *Paenibacillus prosopidis*  *Streptomyces europaeiscabiei*  *Streptomyces nojiriensis*  *Streptomyces subrutilus*  *Streptomyces avidinii*  *Streptomyces lavendulae*  *Streptomyces spororaveus*  *Streptomyces virginiae*  *Streptomyces xanthophaeus* | *Arthrobacter oryzae*  *Pseudarthrobacter oxydans*  *Pseudarthrobacter siccitolerans*  *Flavobacterium fluvii*  *Lentzea aerocolonigenes*  *Streptomyces caeruleatus*  *Streptomyces canus*  *Streptomyces lincolnensis*  *Streptomyces turgidiscabies* | *Pseudomonas corrugata**  *Pseudomonas thivervalensis**  *Pseudomonas putida***  *Streptomyces caeruleatus*  *Streptomyces canus*  *Streptomyces europaeiscabiei*  *Streptomyces lincolnensis*  *Streptomyces turgidiscabies** | *Arthrobacter oryzae*  *Dactylosporangium luteum**  *Pseudarthrobacter oxydans*  *Pseudarthrobacter siccitolerans*  *Corynebacterium variabile*  *Oryzihumus leptocrescens*  *Pseudomonas fluorescens*  *Pseudomonas jessenii*  *Pseudomonas putida*  *Pseudomonas synxantha*  *Streptomyces nojiriensis**  *Streptomyces anulatus*  *Streptomyces avidinii**  *Streptomyces lavendulae**  *Streptomyces rishiriensis*  *Streptomyces spororaveus**  *Streptomyces subrutilus**  *Streptomyces virginiae**  *Streptomyces xanthophaeus** | *Arthrobacter oryzae*  *Dactylosporangium luteum*  *Pseudarthrobacter oxydans*  *Pseudarthrobacter siccitolerans***  *Peribacillus simplex*  *Oryzihumus leptocrescens*  *Streptomyces avidinii*  *Streptomyces caeruleatus*  *Streptomyces lavendulae*  *Streptomyces lincolnensis*  *Streptomyces nojiriensis*  *Streptomyces spororaveus*  *Streptomyces subrutilus*  *Streptomyces turgidiscabies*  *Streptomyces virginiae*  *Streptomyces xanthophaeus*  *Umezawaea tangerina* |
| **Soil associated bacteria** | *Achromobacter xylosoxidans*  *Arthrobacter globiformis*  *Bacillus aryabhattai*  *Bacillus megaterium*  *Bacillus senegalensis**  *Paenisporosarcina indica*  *Peribacillus simplex*  *Pseudarthrobacter scleromae*  *Rhodococcus opacus*  *Rhodococcus wratislaviensis*  *Streptococcus pneumoniae* | *Arthrobacter crystallopoietes**  *Arthrobacter oryzae***  *Nocardia cummidelens*  *Nocardia soli*  *Pseudarthrobacter oxydans***  *Pseudarthrobacter siccitolerans**  *Pseudomonas fluorescens***  *Pseudomonas jessenii***  *Pseudomonas synxantha***  *Saccharomonospora viridis***  *Streptomyces anulatus*  *Streptomyces pratensis*  *Thermobifida fusca*** | *Clostridium vincentii**  *Lentzea kentuckyensis*  *Peribacillus simplex**  *Rhodococcus fascians***  *Streptomyces avidinii*  *Streptomyces lavendulae*  *Streptomyces nojiriensis*  *Streptomyces spororaveus*  *Streptomyces subrutilus*  *Streptomyces virginiae*  *Streptomyces xanthophaeus* | *Arthrobacter oryzae***  *Pseudarthrobacter oxydans***  *Pseudarthrobacter siccitolerans***  *Peribacillus simplex*  *Streptomyces anulatus*  *Streptomyces pratensis* | *Actinosynnema mirum*  *Intrasporangium calvum*  *Lentzea aerocolonigenes*  *Microvirga ossetica*  *Streptomyces anulatus*  *Streptomyces caeruleatus*  *Streptomyces canus*  *Streptomyces europaeiscabiei*  *Streptomyces lincolnensis*  *Streptomyces pratensis* | *Archangium gephyra*  *Bacillus aryabhattai***  *Bacillus megaterium***  *Bacillus senegalensis*  *Bacillus subtilis***  *Microvirga ossetica*  *Streptomyces anulatus*  *Streptomyces pratensis*  *Streptomyces rishiriensis* |

Supplementary Material 2. Species found associated with rhizosphere (ratio >1) or with unplanted soil (ratio <1). Species significantly more abundant in rhizosphere were calculated with Shapiro-Wilk and Student’s test (*p<0.05; **p<0.01). Only species with a relative abundance greater than 1% were retained in the analysis.

|  | **Carvin** | **Brouckerque** | **Eplessier** | **Gouy-Saint-Andre** | **Hallencourt** | **Urvillers** |
| --- | --- | --- | --- | --- | --- | --- |
| **Rhizosphere associated fungi** | *Apiotrichum dulcitum*  *Exophiala salmonis*  *Fusarium flocciferum*  *Fusarium torulosum*  *Fusicolla merismoides*  *Gibberella tricincta*  *Plectosphaerella cucumerina*  *Plectosphaerella oligotrophica*  *Solicoccozyma aeria*  *Thanatephorus cucumeris*  *Trichosporon dulcitum* | *Colletotrichum coccodes*  *Colletotrichum nigrum*  *Exophiala salmonis*  *Mortierellaceae zonata*  *Penicillium canescens*  *Penicillium echinatum*  *Penicillium janczewskii*  *Penicillium jensenii*  *Penicillium murcianum*  *Penicillium radiatolobatum*  *Pseudeurotium bakeri*  *Pseudeurotium hygrophilum*  *Scytalidium lignicola*  *Stachybotrys chlorohalonata*  *Stachybotrys phaeophialis*  *Stachybotrys subreniformis* | *Alternaria infectoria*  *Clonostachys rosea*  *Penicillium canescens***  *Penicillium echinatum***  *Penicillium janczewskii***  *Penicillium jensenii***  *Penicillium murcianum***  *Penicillium radiatolobatum***  *Phoma multirostrata*  *Scytalidium lignicola*  *Talaromyces pinophilus*  *Talaromyces verruculosus* | *Alternaria infectoria*  *Epicoccum nigrum*  *Exophiala salmonis* | *Exophiala salmonis*  *Mortierellaceae zonata*  *Paraphaeosphaeria sporulosa*  *Pseudeurotium bakeri*  *Pseudeurotium hygrophilum*  *Solicoccozyma aeria* | *Acremonium pilosum*  *Apiotrichum dulcitum*  *Chordomyces antarcticus*  *Cladosporium angustisporum*  *Cladosporium anthropophilum*  *Cladosporium cladosporioides*  *Cladosporium funiculosum*  *Cladosporium halotolerans*  *Cladosporium inversicolor*  *Cladosporium lycoperdinum*  *Cladosporium perangustum*  *Cladosporium pseudocladosporioides*  *Cladosporium subuliforme*  *Clonostachys rosea*  *Eucasphaeria capensis*  *Penicillium canescens*  *Penicillium echinatum*  *Penicillium janczewskii*  *Penicillium jensenii*  *Penicillium murcianum*  *Penicillium radiatolobatum*  *Scytalidium lignicola*  *Stephanonectria keithii*  *Tausonia pullulans*  *Thanatephorus cucumeris* |
| **Soil associated fungi** | *Cladosporium allicinum***  *Cladosporium cladosporioides***  *Cladosporium floccosum***  *Cladosporium herbarum***  *Cladosporium limoniforme***  *Cladosporium macrocarpum***  *Cladosporium ossifragi***  *Cladosporium puyae***  *Cladosporium ramotenellum***  *Cladosporium sinuosum***  *Cladosporium tenellum***  *Gibberella intricans*  *Gibberella zeae*  *Gibellulopsis nigrescens*  *Scytalidium lignicola*  *Tausonia pullulans* | *Clonostachys rosea*  *Gibberella avenacea*  *Gibberella tricincta*  *Plectosphaerella cucumerina*  *Scedosporium prolificans*  *Solicoccozyma aeria* | *Acremonium rutilum***  *Cladosporium angustisporum*  *Cladosporium anthropophilum*  *Cladosporium cladosporioides*  *Cladosporium funiculosum*  *Cladosporium halotolerans*  *Cladosporium inversicolor*  *Cladosporium lycoperdinum*  *Cladosporium perangustum*  *Cladosporium pseudocladosporioides*  *Cladosporium* *subuliforme*  *Exophiala salmonis*  *Mortierellaceae zonata**  *Pseudeurotium bakeri**  *Pseudeurotium hygrophilum**  *Solicoccozyma aeria*  *Stachybotrys chlorohalonata***  *Stachybotrys phaeophialis***  *Stachybotrys subreniformis*** | *Apiotrichum dulcitum*  *Cladosporium angustisporum*  *Cladosporium anthropophilum*  *Cladosporium cladosporioides*  *Cladosporium funiculosum*  *Cladosporium halotolerans*  *Cladosporium inversicolor*  *Cladosporium lycoperdinum*  *Cladosporium perangustum*  *Cladosporium pseudocladosporioides*  *Cladosporium subuliforme*  *Mortierellaceae zonata*  *Scytalidium lignicola*  *Tausonia pullulans*  *Trichosporon dulcitum* | *Apiotrichum dulcitum*  *Cladosporium angustisporum*  *Cladosporium anthropophilum*  *Cladosporium cladosporioides*  *Cladosporium funiculosum*  *Cladosporium halotolerans*  *Cladosporium inversicolor*  *Cladosporium lycoperdinum*  *Cladosporium perangustum*  *Cladosporium pseudocladosporioides*  *Cladosporium subuliforme*  *Clonostachys rosea*  *Penicillium canescens*  *Penicillium echinatum*  *Penicillium janczewskii*  *Penicillium jensenii*  *Penicillium murcianum*  *Penicillium radiatolobatum*  *Scytalidium lignicola*  *Tausonia pullulans* | *Arthopyrenia salicis*  *Exophiala salmonis*  *Fusarium solani*  *Fusarium venenatum*  *Mortierellaceae zonata*  *Solicoccozyma aeria* |
