# Supplementary material for: Unveiling the hidden allies of industrial chicory—a metagenomic exploration of rhizosphere microbiota and their impact on productivity and plant health
Source: Front Microbiol. 2025 May 9;16:1509094. doi: 10.3389/fmicb.2025.1509094 (PMC12098591; doi:10.3389/fmicb.2025.1509094)
Supplement: Supplementary file 1 [file Data_Sheet_1.zip › Supplementary Material 1.docx]

| Plot | Sample | | [DNA] ng/μL | A260/A280 | A260/A230 |
| --- | --- | --- | --- | --- | --- |
| Carvin | Bulk soil | 1 | 166.1 | 1.5 | 1.85 |
|  |  | 2 | 160.1 | 1.52 | 1.97 |
|  |  | 3 | 193.3 | 1.52 | 1.93 |
|  |  | 4 | 186.7 | 1.6 | 1.76 |
|  |  | 5 | 165.6 | 1.51 | 1.56 |
|  | Rhizosphere | 1 | 173.2 | 1.55 | 1.78 |
|  |  | 2 | 163.3 | 1.53 | 1.93 |
|  |  | 3 | 150.6 | 1.52 | 1.85 |
|  |  | 4 | 154.5 | 1.57 | 1.83 |
|  |  | 5 | 191.1 | 1.53 | 1.92 |

Supplementary Material 1. Amount and quality of DNA in samples from all plots. Absorbance was measured at 260nm (A260) for nucleic acids, 280nm (A280) for proteins, and 230nm (A230) for residual chemicals.

| Plot | Sample | | [DNA] ng/μL | A260/A280 | A260/A230 |
| --- | --- | --- | --- | --- | --- |
| Brouckerque | Bulk soil | 1 | 231.5 | 1.41 | 1.6 |
|  |  | 2 | 243.7 | 1.72 | 1.91 |
|  |  | 3 | 212.2 | 1.75 | 2.09 |
|  |  | 4 | 191.4 | 1.79 | 1.66 |
|  |  | 5 | 198.5 | 1.77 | 1.61 |
|  | Rhizosphere | 1 | 159.6 | 1.5 | 1.6 |
|  |  | 2 | 136.6 | 1.47 | 1.86 |
|  |  | 3 | 211.3 | 1.81 | 1.55 |
|  |  | 4 | 162.3 | 1.91 | 1.8 |
|  |  | 5 | 146.4 | 1.26 | 1.44 |

| Plot | Sample | | [DNA] ng/μL | A260/A280 | A260/A230 |
| --- | --- | --- | --- | --- | --- |
| Eplessier | Bulk soil | 1 | 272 | 1.53 | 2.04 |
|  |  | 2 | 249.6 | 1.52 | 1.95 |
|  |  | 3 | 213.2 | 1.46 | 2.02 |
|  |  | 4 | 262 | 1.49 | 1.97 |
|  |  | 5 | 193.8 | 1.48 | 1.44 |
|  | Rhizosphere | 1 | 239.3 | 1.5 | 1.7 |
|  |  | 2 | 229.7 | 1.5 | 1.82 |
|  |  | 3 | 226.4 | 1.49 | 1.95 |
|  |  | 4 | 193.5 | 1.44 | 2.07 |
|  |  | 5 | 223.7 | 1.49 | 1.92 |

| Plot | Sample | | [DNA] ng/μL | A260/A280 | A260/A230 |
| --- | --- | --- | --- | --- | --- |
| Gouy-Saint-Andre | Bulk soil | 1 | 200.6 | 1.52 | 1.71 |
|  |  | 2 | 184.4 | 1.5 | 1.52 |
|  |  | 3 | 221.6 | 1.54 | 1.75 |
|  |  | 4 | 264.6 | 1.57 | 1.78 |
|  |  | 5 | 145 | 1.54 | 2 |
|  | Rhizosphere | 1 | 188.1 | 1.55 | 1.41 |
|  |  | 2 | 195.7 | 1.48 | 2.45 |
|  |  | 3 | 167.3 | 1.52 | 1.28 |
|  |  | 4 | 133.6 | 1.58 | 1.78 |
|  |  | 5 | 111 | 1.45 | 1.88 |

| Plot | Sample | | [DNA] ng/μL | A260/A280 | A260/A230 |
| --- | --- | --- | --- | --- | --- |
| Hallencourt | Bulk soil | 1 | 274.6 | 1.48 | 1.99 |
|  |  | 2 | 244 | 1.54 | 1.9 |
|  |  | 3 | 275.8 | 1.49 | 1.86 |
|  |  | 4 | 321.9 | 1.61 | 1.76 |
|  |  | 5 | 272.5 | 1.49 | 1.58 |
|  | Rhizosphere | 1 | 214.4 | 1.49 | 1.79 |
|  |  | 2 | 277 | 1.51 | 1.95 |
|  |  | 3 | 258.6 | 1.54 | 1.98 |
|  |  | 4 | 234.4 | 1.46 | 2.12 |
|  |  | 5 | 243.5 | 1.51 | 1.84 |

| Plot | Sample | | [DNA] ng/μL | A260/A280 | A260/A230 |
| --- | --- | --- | --- | --- | --- |
| Urvillers | Bulk soil | 1 | 225.8 | 1.51 | 1.84 |
|  |  | 2 | 246.3 | 1.54 | 1.97 |
|  |  | 3 | 187.7 | 1.54 | 2.21 |
|  |  | 4 | 228.4 | 1.5 | 2.04 |
|  |  | 5 | 221.1 | 1.49 | 1.88 |
|  | Rhizosphere | 1 | 169.9 | 1.52 | 1.44 |
|  |  | 2 | 233.4 | 1.7 | 2 |
|  |  | 3 | 219.1 | 1.51 | 1.86 |
|  |  | 4 | 207.7 | 1.52 | 2.05 |
|  |  | 5 | 233.4 | 1.56 | 1.88 |
